# Key points of reducing neurologic complications in frozen elephant trunk technique

**DOI:** 10.5935/1678-9741.20150056

**Published:** 2015

**Authors:** Murat Kadan, Gokhan Erol, Kubilay Karabacak, Mevlüt Kobuk

**Affiliations:** Gulhane Military Academy of Medicine, Etlik Ankara, Turkey.

Dear editor,

We have read the interesting article entitled "Surgical treatment of complex aneurysms and thoracic aortic dissections with the Frozen Elephant Trunk technique" carefully^[1]^. The authors report their initial experience with this technique in 21 patients. First of all we appreciated the authors for this nice study. We would like to add some critics about this study.

There were some neurologic complications such as stroke (in one patient) and paraplegia (in two patients) in the study. Did the authors make any assessment about neurologic complications and their protection strategies? This is a very important point that should be detailed in paper. The exact mechanism of spinal cord injury in frozen elephant trunk interventions is not fully understood. Stent graft length, thromboembolism, and spinal cord ischemia time during total circulatory arrest are considered responsible factors^[2]^. Cerebrospinal fluid drainage is recommended for spinal cord protection strategy in current guideline (Class I, level of evidence B)^[3]^. Proximal aortic pressure maintenance and distal aortic perfusion are some of the other recommendations (Class IIa, level of evidence B). From this point, did the authors use any of suggested protection method?

On the other hand, neurologic complications can also be associated with distal length of endovascular prosthesis. In literature, 130 mm stent length is recommended for preventing paraplegia^[2]^. What was the distal length of prosthesis in these patients? Did authors make any assessment about distal position of stent in patients with neurologic complications?

The authors performed surgery in conventional operating room, without the use of scopes or guidewire. How can authors identify the true lumen? Wasn't it a risk? Can mentioned neurologic complications as well as renal failure be associated with possible selection of incorrect lumen? Why didn't authors use guidewire? Has the dissection also included both femoral arteries? Hybrid operating room doesn't exist in many centers, however, guidewire may be used to identify true lumen. In our center, we also don't have hybrid operating room, but we routinely use guidewire from intact femoral artery through descending thoracic aorta in retrograde way. Therefore we are able to see the true lumen directly.

In conclusion, we consider that, this single stage technique is so useful especially in complex aortic pathologies. Learning curve is a reality of these novel strategies of course, but morbidity rates can be decreased with appropriate surgical strategies and known guideline recommendations.

**Murat Kadan, MD; Gokhan Erol, MD; Kubilay Karabacak, MD; Mevlüt Kobuk,
MD;** **Gulhane Military Academy of Medicine, Etlik Ankara, Turkey.**
